# Assessing skeletal maturity in a UK modern female population

**DOI:** 10.1007/s12024-025-01044-1

**Published:** 2025-08-06

**Authors:** Marta San-Millán, Trish Holmes, Stefano De Luca, Lucina Hackman, Nicholas Márquez-Grant

**Affiliations:** 1 Medical Sciences Department, Clinical Anatomy, Embryology and Neuroscience Research Group (NEOMA), Faculty of Medicine, University of Girona, Girona, 17071 Spain; 2 Healthcare Specialist, Public Health England, London, UK; 3 Organisms and Systems Biology, Faculty of Biology, University of Oviedo, 33006 Oviedo, Spain; 4 Centre for Anatomy and Human Identification (CAHID), University of Dundee, Dundee, DD1 4HN UK; 5 Cranfield Forensic Institute, Cranfield University, College Road, Bedford, M43 0AL UK

**Keywords:** Age estimation, Skeletal age, Greulich and Pyle, Hand and wrist x-ray, Legal medicine

## Abstract

**Objective:**

The study aimed to examine the accuracy of Greulich and Pyle (GP) methodology for estimating age in a female UK sample since there is a dearth of studies testing its applicability in the UK.

**Materials and methods:**

Radiographs from the left wrist and hand belonging to 257 female individuals from two different hospitals from the UK ranging from 10 to 17 years old were analysed. Correlation was performed between skeletal age (SA) and chronological age (CA) values as well as mean comparisons. In addition, the mean difference (MD) and mean absolute difference (MAD) between these two parameters were also examined to study inaccuracy values.

**Results:**

A strong correlation was found between SA and CA (*r* = 0.887; *p* < 0.001). The MD between SA and CA is positive (0.51 ± 12.43 months), meaning that SA based on Greulich and Pyle atlas overestimates CA in general terms (minimum: − 40.17 months; maximum: 31.13 months). In the case of MAD, the mean value is 9.96 ± 7.44 months (minimum: 0.07 months; maximum: 40.17 months). In addition, no significant differences were found between SA and CA either in the global sample or in most of the age cohorts, with two exceptions: 14-year-olds, where mean SA was significantly higher than CA, and 16-year-olds, where the opposite results were observed.

**Conclusion:**

These results suggest that the GP atlas can be applied to the present female UK population, although caution must be taken when applied to estimating the age of post-pubertal UK girls.

## Introduction

In the context of age estimation, chronological age (CA) is defined as the actual age of an individual, calculated from the date of birth, and is used as the reference standard for evaluating biological development [1]. In contrast, skeletal age (SA) refers to the degree of skeletal maturation, typically assessed through radiographic analysis of the hand and wrist [1]. The estimation of SA through imaging is a diagnostic tool frequently used for the evaluation of certain disorders, specifically to monitor response to medical therapy and to determine the growth potential of children [2]. However, age estimation is also necessary for medico-legal cases where undocumented minors are involved or where there are doubts about someone’s age. Despite the existence of international protocols regarding the point at which an individual moves from being considered a child, to being considered an adult (18 years) [3], there is still no unified protocol in Europe for age estimation [4]. In fact, although both the Study Group on Forensic Age Diagnostics of the German Association of Forensic Medicine (AGFAD) and the European Union Agency for Asylum (EUAA) continue to work on unifying best practices and protocols at European level, in the end each member country acts independently and carries out its own protocols for estimating age. Very often, the recommended holistic procedures are not applied, i.e. not all anatomical districts recommended for estimating age are analysed (hand-wrist, third molar and clavicle [5]), and even more serious is the fact that, in several countries, the analysis of ossification of the wrist bones is used exclusively. Finally, statistical procedures lack adequate tests to quantify the uncertainty of the results. All of this means that there are very significant technical and ethical errors in processes that can lead to misclassification of individuals, creating legal uncertainties for the alleged migrant minors without family references [6].

Although forensic medicine and forensic anthropology typically focus on the 18-year threshold, and the term ‘minor’ is mostly used in civil or criminal codes describing all children below 18 years, the pre-adolescent and adolescent periods are also meaningful in clinical [7–9], competitive sports [10, 11] and medicolegal contexts [12].

In all European Union (EU) Member States, the age of majority is 18 years, except for Scotland, where children are considered to have full legal capacity from the age of 16 years. At 16, individuals can obtain a National Insurance number, drive a moped, and engage in some activities that require parental permission at younger ages. In England and Wales, children can be held liable for criminal offences from the age of 10 [13]. In general, the cut-off age of 16 years is indeed important in the United Kingdom (UK) in some domains such as medical consent and the age of sexual consent. Age assessment can assist with welfare allocation, education levels and in females in particular, assist the victim in cases of sexual exploitation, forced labour, domestic servitude, potential rape, underage marriage, etc [14]. Although cases involving males are prevalent in medico-legal contexts, there has been a recent increase in the number of cases involving females that require age estimation; for example, a 2011–2018 study from Barcelona (Spain) demonstrated that females requiring age estimation increased in number from 1.2% (2011) to 5.8% (2014) [15]. In addition, Thicot et al. reported 5.4% female cases between 2010 and 2022 from the University Centre of Legal Medicine Lausanne-Geneva, Switzerland [16].

Among the different available reference datasets that can be utilised to assess the degree of skeletal development, Greulich and Pyle (GP) based on the left hand and wrist is one of the most widely used in clinical practice worldwide [17]. The atlas was originally developed to assess skeletal development in children and adolescents with known CA, and in this capacity, it has been routinely implemented in paediatric radiology. It has been more recently utilized as a method of estimating age from SA in medico-legal or forensic cases. The GP method for age estimation involves a qualitative assessment in which a radiograph of the subject’s left hand and wrist is visually compared to a series of standard reference plates in the GP atlas. Each plate represents the median skeletal development for a specific chronological age and sex. The atlas was developed using radiographs from 1,000 males and 1,000 females, primarily of European-American ancestry, collected between 1931 and 1942 at the Brush Foundation Study in Cleveland, Ohio. The evaluator selects the reference image that most closely matches the subject’s radiograph in terms of ossification centers, epiphyseal fusion, and bone morphology. The SA is then assigned based on the age associated with the selected reference image. This approach relies on expert judgment and assumes a relatively linear and predictable pattern of skeletal maturation, which may not hold uniformly across all populations or individuals. Considering the limited representativeness of the original reference sample - both historically and demographically - from which the GP atlas was developed, assessing its validity and reliability in contemporary and ethnically diverse populations remains essential [1, 18–20]. The applicability of the atlas has been extensively tested in recent years in several countries worldwide: e.g [21–36]. In the UK, despite the creation of a recent *Interim Age Estimation Science Advisory Committee* [37], studies regarding the validity of this method today are limited [32, 36]. Only two studies have assessed the applicability of the GP method in the UK to date. One was conducted in Scotland [32], and the other in England [36]. The former may not fully represent the English population, and the latter included individuals only up to 15 years of age. Notably, and to the best of our knowledge, the study presented here represents the first analysis for females who are older than 15 years conducted in England.

The aim of this study is to evaluate the accuracy of the GP methodology in estimating CA in a sample of modern UK female individuals aged 10 to 17 years, with a particular focus on its effectiveness in determining whether an individual has reached the legal age of 16 years in an English population.

## Materials and methods

A cross-sectional study was performed examining radiographic images of the left hand and wrist of 257 individuals aged 10 to 17 years (mean = 13.37; SD = 2.10; minimum 10.03 years old; maximum = 16.99 years old) (Table 1). These were obtained from Great Ormond St Children’s Hospital (GOSH) in London (*n* = 120; mean = 13.15 years; SD = 1.98) and The Royal Alexandra Children’s Hospital (RACH) in Brighton (*n* = 137; mean = 13.55 years; SD = 2.19), both located in England, UK. This age range was selected because it includes the important and mentioned 16-year threshold recognised in the UK, and the legal age of criminal responsibility, which begins at 10 years old. Statistically, the study wanted to exclude individuals under the age of 10 so as not to cause common biases such as underestimating their age [36]. Individuals older than 17 years were also left out because, in the pediatric hospitals involved in the study, there was not a statistically significant number of subjects to include in the final sample analyzed. This is due to the fact that individuals of that age can be treated either in the aforementioned pediatric hospitals or directly in an adult hospital.

**Table 1:** Number of radiographic images by age group

Chronological age group	*N*
10–10.99 years	48
11–11.99 years	24
12–12.99 years	57
13–13.99 years	18
14–14.99 years	48
15–15.99 years	15
16–16.99 years	47
**TOTAL**	**257**

GOSH is an international centre of excellence in child healthcare with over 240,000 patients each year mostly referred from other UK hospitals and overseas. Data collection was undertaken in 2015, and female left hand and wrist radiographs were retrieved from the PACS (patient archiving and communication system) that had been taken from patients attending GOSH between January 2011 and December 2014. RACH covers the area of Brighton and Hove. The radiographs selected for this study were taken as part of diagnostic tests of female children and adolescents aged between 10 and 17 years old who attended the Children’s Emergency Department at RACH between January 2008 and May 2015. Information on the biological sex, date of birth, date when the radiograph was taken, and side of body was available for every individual and in all cases the radiographs were anonymised. In both cases, radiographs from patients who had been subject to referrals for the investigation of endocrine disorders were excluded from the study, as were those with osteogenesis imperfecta, fracture at the site of the epiphysis or where individuals were described in the medical records as underweight, stunted and/or with leg length discrepancy. However, requests for ‘bone-age’ radiographs in order to assess growth prior to epiphysiodesis (a surgical procedure) were used. Permission to access and publish the data from the hospitals was approved by the NHS and this study was approved through Cranfield University Research Ethics System (Ref. CURES/25984/2025).

The observer (TH) examined each radiographic image without knowledge of the CA and assigned a skeletal age using the GP atlas [17]. The methodology involved identifying the closest match between the radiograph and the images within the atlas. Both CA and SA were converted from years into months for the purpose of improving accuracy and to enhance the precision of the statistical analyses. All the data were compiled by the second author (TH), with support when required from a Consultant Radiologist as well as the last author (NMG), a forensic anthropologist.

Mean values of SA and CA were statistically compared by paired sample t-test analyses, both in the global sample and according to year cohorts. In addition, a Pearson correlation analysis was carried out between CA and SA. To quantify the difference between the GP-based assigned SA and the CA, the CA of each individual was then subtracted from the SA. A positive value indicated that an individual’s age had been overestimated, i.e. an age older than their actual CA; whilst a negative value meant that an individual’s age had been underestimated, i.e. an age younger than their actual CA. In addition, the absolute difference between the SA and CA was also calculated for each age cohort to understand the discordance regardless of the error direction.

Student T-test or U Mann-Whitney test were employed to compare the mean CA, the mean difference (MD) and the mean absolute difference (MAD) between the CA and the SA of both UK samples. Lastly, to investigate if significant differences were present in the MD and MAD between SA and CA across age cohorts, ANOVA or Kruskal-Wallis analyses with corresponding post-hoc tests were performed.

All the statistical analyses were performed by IBM SPSS 29.0 Statistics and the results were considered significant at *p* < 0.05.

## Results

Table 2 summarises the global results of the present study. The London group demonstrated differences between SA and CA ranging from an underestimation of 36.10 months (≈ 3.01 years) to an overestimation of 27.63 months (≈ 2.30 years). The Brighton population, on the other hand, showed differences ranging from an underestimation of 40.17 months (≈ 3.35 years) to an overestimation of 31.13 months (≈ 2.59 years). However, there were no significant differences between the mean inaccuracy values (MD between SA and CA) of the London (0.08 ± 12.99 months) and Brighton (0.89 ± 11.96 months) samples (U = 7774.5; *p* = 0.454). Regarding the absolute difference between SA and CA, MAD was 10.53 ± 7.54 months in the London sample and 9.46 ± 7.34 months in the Brighton one; however no significant differences were found between the samples (t = -1.156; fd = 255; *p* = 0.124). Since no significant differences between mean CA (t = 1.515; fd = 255; *p* = 0.065), MD, or MAD were found between the London and Brighton samples, the global sample was analysed as a whole to achieve higher sample size and statistical robustness.

**Table 2:** Descriptive of mean difference (top) and mean absolute difference (bottom) between skeletal age (SA) and chronological age (CA) in months

Difference						
Sample	Mean	SD	95% CI	Minimum	Maximum	*N*
London	0.08	12.99	−2.27–2.42	− 36.10	27.63	120
Brighton	0.89	11.96	−1.13–2.91	− 40.17	31.13	137
Global	0.51	12.43	−1.02–2.04	−40.17	31.13	257
Absolute difference						
London	10.53	7.54	9.17–11.89	0.23	36.10	120
Brighton	9.46	7.34	8.21–10.70	0.07	40.17	137
Global	9.96	7.44	9.04–10.87	0.07	40.17	257

**Fig. 1:**
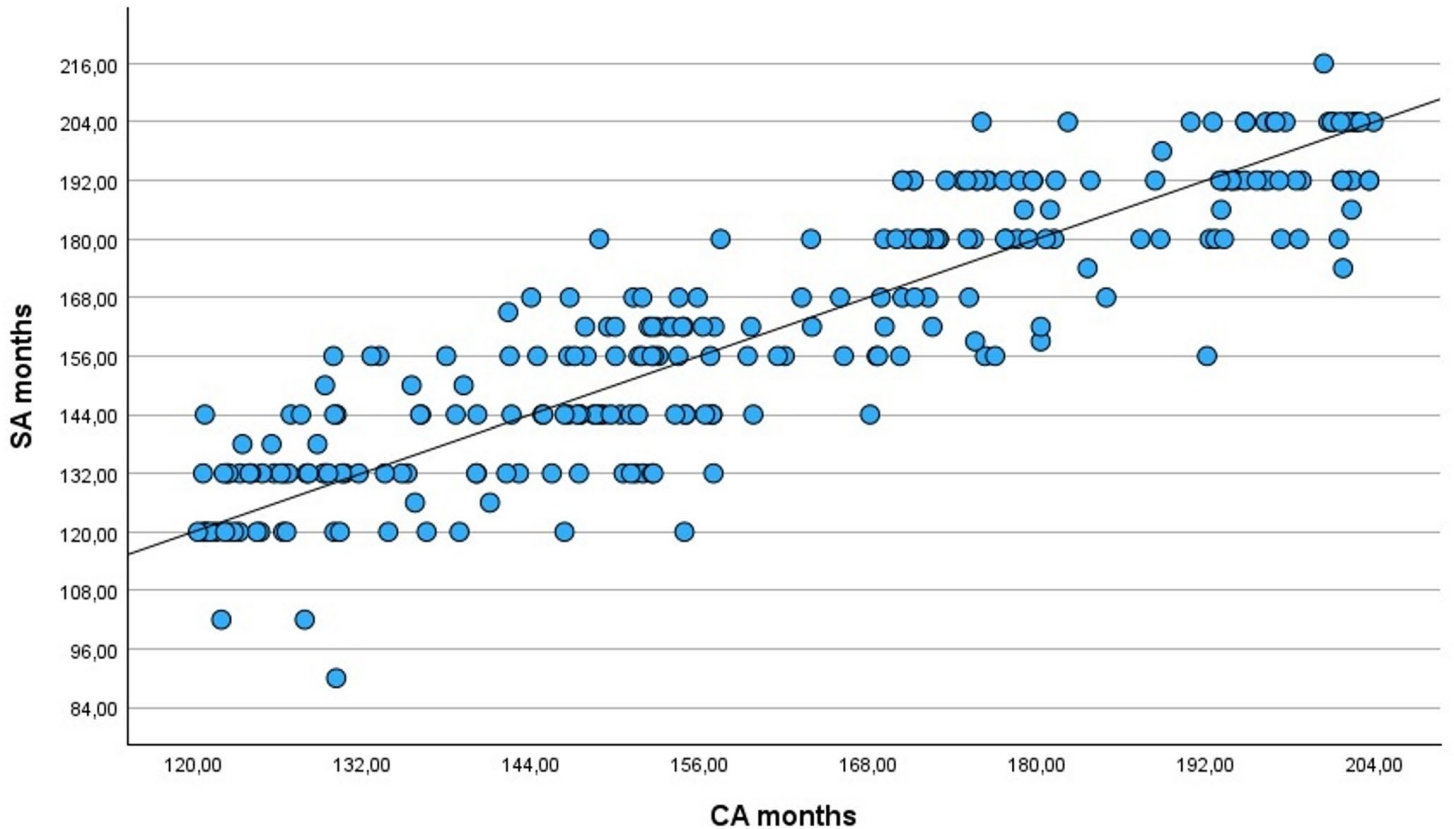
Pearson correlation between chronological age (CA) and skeletal age (SA) in the global sample

As shown in Table 2, the MD between SA and CA is positive (0.51 ± 12.43 months), meaning that GP overestimates CA in general terms, ranging from underestimating by 40.17 months (≈ 3.35 years) to overestimating by 31.13 months (≈ 2.59 years). In the case of MAD between SA and CA, the mean value is 9.96 ± 7.44 months, ranging from 0.07 months (≈ 2.17 days) to 40.17 months (≈ 3.35 years). In addition, a strong correlation was found between CA and SA (*r* = 0.887; *p* < 0.001) in the global sample (Fig. 1).

Table 3 displays age-based information about the mean comparison between CA and SA. No significant differences were found in the global sample and in most of the age cohorts, except for 14- and 16-year-olds subjects: in the former, mean SA was significantly higher than CA while the opposite result was rendered in the latter.

**Table 3:** Paired sample t-test analyses between chronological age (CA) and skeletal age (SA) in years by year cohorts in the global sample. GP = Greulich and Pyle methodology. Significant values are marked in bold

Chronological age groups	CA mean ± SD	SA (GP) mean ± SD	t	*p* - value	*N*
10–10.99 years	10.48 ± 0.29	10.72 ± 1.01	-1.657	0.104	48
11–11.99 years	11.50 ± 0.28	11.62 ± 1.09	-0.486	0.631	24
12–12.99 years	12.53 ± 0.27	12.51 ± 1.10	0.172	0.864	57
13–13.99 years	13.34 ± 0.29	13.11 ± 1.05	1.001	0.331	18
14–14.99 years	14.48 ± 0.28	14.89 ± 1.13	-2.743	**0.009**	48
15–15.99 years	15.35 ± 0.31	15.28 ± 1.16	0.253	0.804	15
16–16.99 years	16.51 ± 0.32	16.16 ± 0.90	2.810	**0.007**	47
Total	13.37 ± 2.10	13.41 ± 2.22	-0.652	0.515	257

Age-based results regarding the MD between SA and CA are displayed in Table 4 (top). On average, the MD ranged between − 4.19 ± 10.23 months (16 years cohort) to 4.97 ± 12.54 months (14 years cohort). Thus, MD were overall positive (overestimation) in the 10-, 11- and 14-year-old cohorts while they were negative (underestimation) in the 12-, 13-, 15- and 16-year age cohorts. These MD were significantly different between year cohorts (KW = 19.293; *p* = 0.004). Post-hoc analyses demonstrated significant differences between two pairs: (1) the 10- (2.82 ± 11.81 months) and 16-year (-4.19 ± 10.23 months) cohorts (*p* = 0.044) and (2) the 14- (4.97 ± 12.54 months) and 16-year (-4.19 ± 10.23 months) cohorts (*p* = 0.003). There was an overestimating in the 14-year-olds. Out of 48 individuals whose chronological age was 14-years, a total of 15 (31.2%) were assigned a SA of 16 years and 1 was estimated as an 18-year-old. By contrast, the tendency was to underestimate the age of 16-year-old subjects. On the other hand, when analysing the MAD between SA and CA (Table 4, bottom), values ranged from 8.12 ± 7.43 months (16-years cohort) to 11.53 ± 6.83 months (14-years cohort). Thus, the lowest MAD were found in the 10-, 13 and 16-year cohorts while the highest were found in the 11-, 12-, 14- and 15-year cohorts. However, these differences were not significant between age groups (F = 1.277; *p* = 0.268).

**Table 4:** Descriptive analysis of the differences (top) and absolute differences (bottom) between skeletal age (SA) and chronological age (CA) in months by year cohorts in the global sample

Difference SA - CA						
Chronological age groups	Mean	SD	95% CI	Minimum	Maximum	*N*
10–10.99 years	2.82	11.81	−0.61–6.25	−40.17	26.03	48
11–11.99 years	1.33	13.29	−4.28– 6.94	−18.93	23.33	24
12–12.99 years	−0.29	13.18	−3.79–3.21	−34.93	31.13	57
13–13.99 years	−2.71	11.57	−8.46–3.04	−25.00	22.50	18
14–14.99 years	4.97	12.54	1.32–8.61	−24.13	27.93	48
15–15.99 years	−0.84	12.64	−7.84–6.16	− 21.27	21.80	15
16–16.99 years	−4.19	10.23	−7.19-−1.19	−36.10	15.60	47
Absolute difference SA - CA						
10–10.99 years	8.85	8.22	6.46–11.23	0.23	40.17	48
11–11.99 years	11.30	6.72	8.47–14.14	1.37	23.33	24
12–12.99 years	10.65	7.65	8.62–12.68	0.83	34.93	57
13–13.99 years	9.19	7.21	5.61–12.78	0.77	25.00	18
14–14.99 years	11.53	6.83	9.55– 13.51	0.60	27.93	48
15–15.99 years	10.37	6.72	6.65–14.10	0.63	21.80	15
16–16.99 years	8.12	7.43	5.93–10.30	0.07	36.10	47

The confidence intervals shown in Tables 2 and 4 were narrow enough to be acceptable given that the radiographs in the GP atlas have age intervals of mainly 12 months for the age groups in this study. The only exception is a reference image for 13 years and 6 months, giving a reduced spacing between images. The GP atlas recommends that a given age is acceptable within two standard deviations to account for natural variation. The standard deviations specified in this analysis are therefore applicable to estimating age in the analysed sample.

**Fig. 2:**
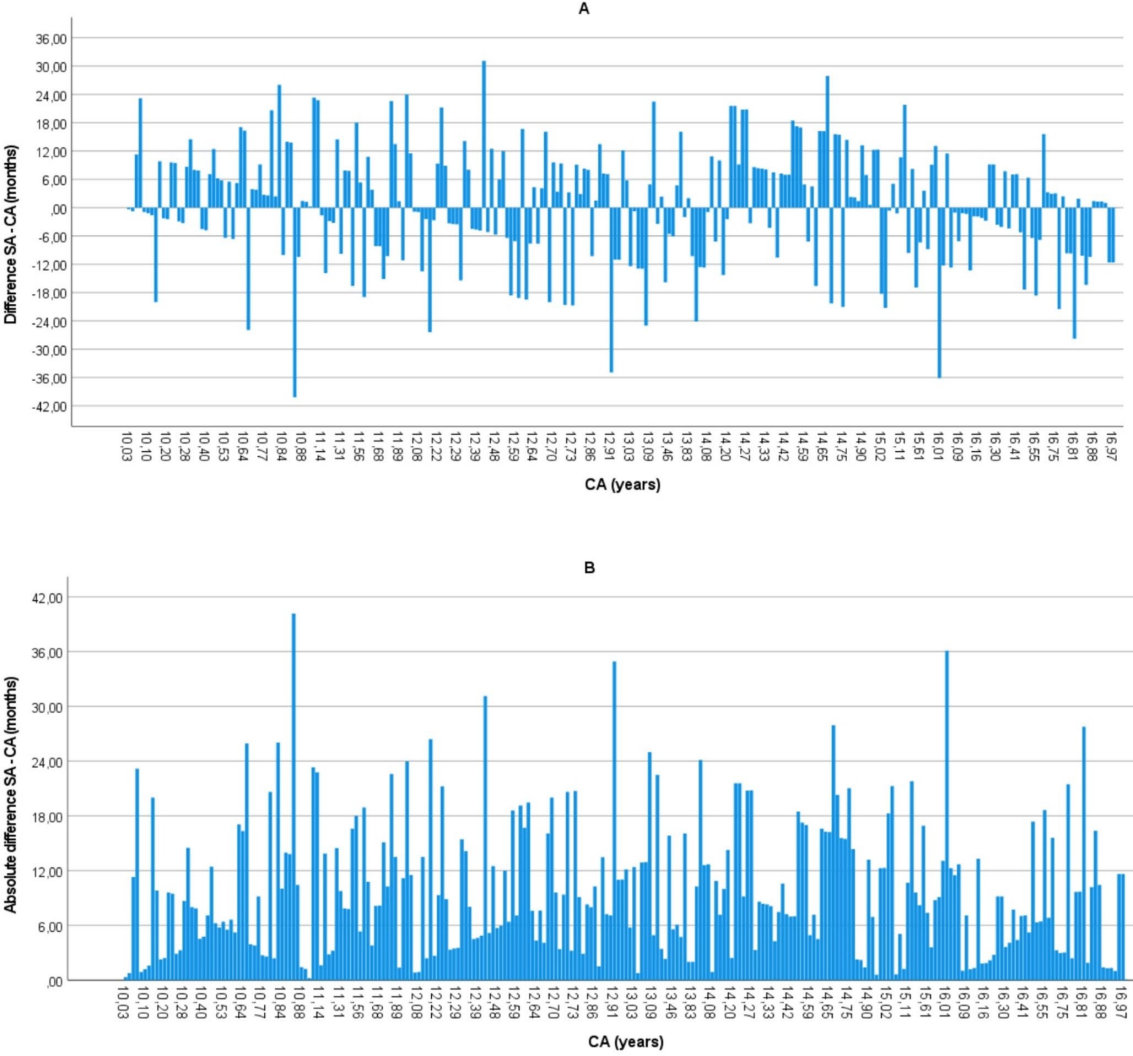
Individual representation of the difference (**A**) and absolute difference (**B**) between skeletal age (SA) and chronological age (CA) in months

Figure 2 displays the difference (2A) and absolute difference (2B) between SA and CA of all individuals in the sample. The graphics do not represent any recognisable trend or progression across CA; however, there are two results that are worth highlighting. First, there are just two individuals with a difference higher than 36 months (≈ 3 years). Here, both results were negative, meaning SA was estimated to be lower than CA. Specifically, one individual from the London sample whose CA was 16 years old was underestimated by 36 months (SA = 13 years old). The second outlier was an individual from Brighton whose CA was 10 years old but was underestimated by 40 months (SA = 7.5 years old). Unfortunately, there was no clinical information available in these cases. The second result to emphasize is that Fig. 2 describes short ascending and descending slopes in the bars, coinciding with the thresholds between year cohorts. Thus, Fig. 2A displays decreasing patterns of overestimation and increasing patterns of underestimation from the start of the year cohort to the end of it. Figure 2B, on the other hand, shows triangular patterns, with less absolute differences at the beginning and end of the year cohorts and highest values in the intermediate positions.

**Fig. 3:**
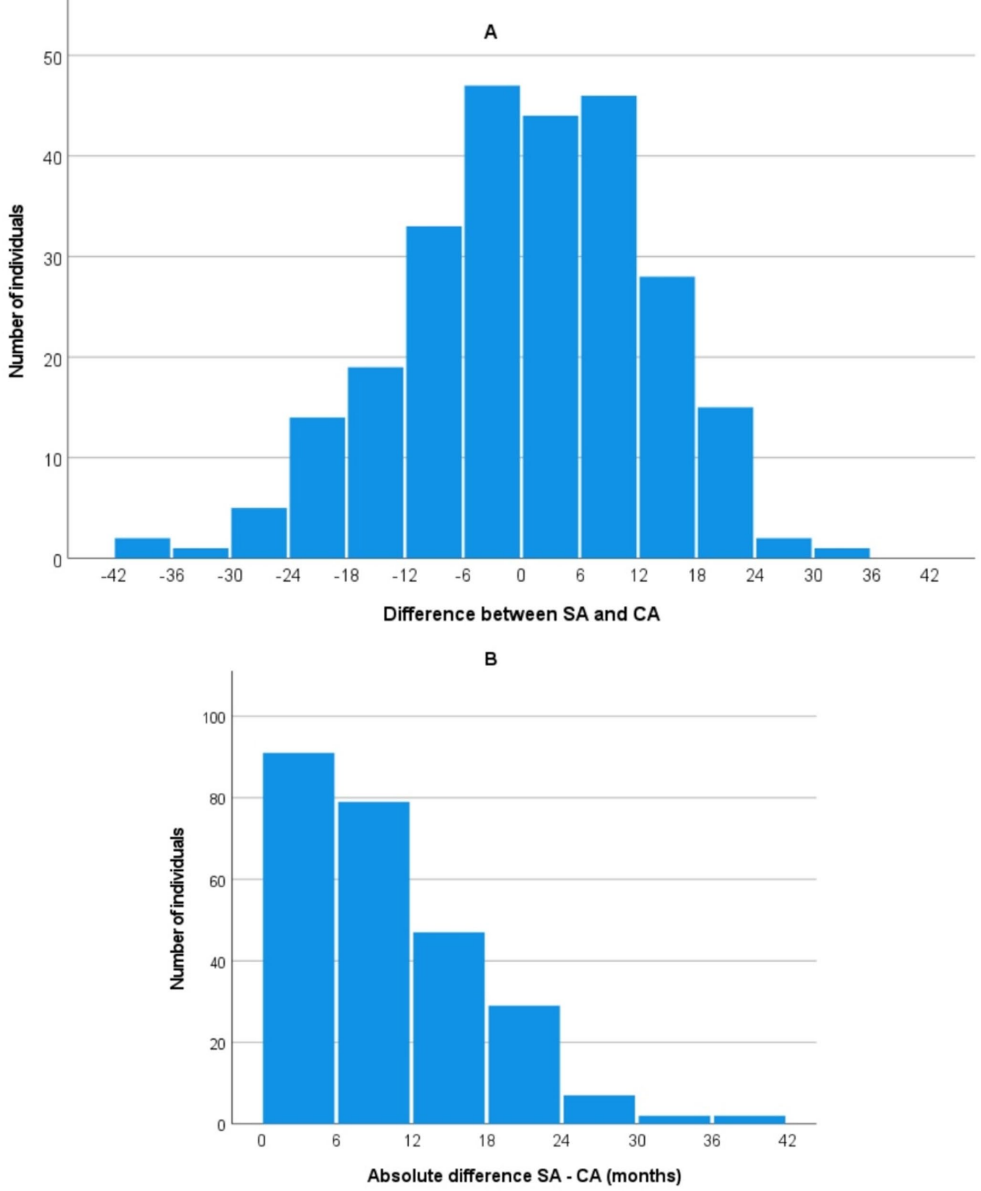
**A**) Distribution of the difference (**A**) and absolute difference (**B**) between skeletal age (SA) and chronological age (CA) using 6-month intervals in the global sample

Figure 3 represents the difference (3A) and absolute difference (3B) between SA and CA separated into 6-month intervals. Data from 3A demonstrates an approximate symmetrical distribution, despite negative values reaching higher scores. The intervals with more individuals involved were underestimated up to 6 months and overestimated up to 1 year. By contrast, Fig. 3B displays absolute differences in the same 6-month intervals, rendering a clear decreasing pattern.

## Discussion

This study assessed the applicability of the Greulich and Pyle’s atlas [17] for age estimation in two female population samples from the UK. Overall results show a strong correlation between SA and CA in the analysed sample, resulting in an MAD between those parameters of 9.96 ± 7.44 months. Other European studies have also reported a strong and significant correlation between SA and CA [21–23, 32], reaching almost perfect values such as 0.974 in the Netherlands [24]. Although a slight overestimation was reported in the current study (0.51 ± 12.43 months), of interest are results found in the 14- and 16-year-old cohorts, with an overestimation in the former and an underestimation in the latter. Similarly, a Canadian study evaluating GP methodology [9] found SA to be significantly higher than CA in the 14-year cohort (9.0 ± 12.24 months). However, they also reported overestimation in the 16-year-old cohort (6.48 ± 9.48 months).

Given the inter-population variation in skeletal maturation [6, 25–28], the applicability of GP atlas to different geographic groups has also been extensively discussed [1, 18]. As Dahlberg and colleagues [1] pointed out, inter-population heterogeneity has been a topic of discussion for decades, including the influence of population affinity [25, 26, 28], nutritional status [38, 39], socioeconomic level [40, 41] or BMI [40, 42, 43]. A study on a Malawian population by Lewis et al. [44] reported socio-economic factors as the major cause of delayed maturation, but also reported malaria and diarrhea as contributing factors.

Table 5 displays the main results from applying GP to female samples from different European countries in the present century. Amongst them are two UK-based publications. The first one, by Hackman and Black [32], analysed the reliability of the GP atlas for a modern Scottish sample of 406 individuals of both sexes between 0 and 21 years old. The second, a study by Alshamrani and Offiah [36] examining 392 radiographs from children of both sexes with ages ranging between 2 and 15 years and comparing CA with the GP and Tanner-Whitehouse (TW3) standards [45] across different socioeconomic categories. In this specific case, BoneXpert, a software developed in 2009 to automatically calculate the maturity stage [46], was used to assign the SA.

**Table 5:** Comparison of MD (mean difference between skeletal and chronological ages) results across literature and the present study when applying Greulich and Pyle methodology in female samples from different European populations

Reference	Origin	Age range (in years)	N	MD (in months) ± SD							
				Global *	10–10.99 years	11–11.99 years	12–12.99 years	13–13.99 years	14–14.99 years	15–15.99 years	16–16.99 years
van Rijn 2001 [24]	The Netherlands	5–20	294	**1.7**	x	x	x	x	x	x	x
Schmidt 2007 [29]	Germany	1–18	303	*-4.68 ± 12.96*	x	x	x	x	x	x	x
Büken 2007 [21]	Turkey	11–19	241	x	x	6.96 ± 11.64	6.6 ± 12.84	4.68 ± 17.4	13.2 ± 11.16	4.68 ± 12.6	11.4 ± 13.56
Büken 2009 [22]	Turkey	11–16	164	**7.92 ± 13.32**	x	6.96 ± 11.64	6.84 ± 13.2	5.88 ± 16.56	13.68 ± 11.04	4.92 ± 12.96	x
Santos 2011 [23]	Portugal	12–20	94	-0.28	x	x	x	x	x	x	x
Cantekin 2012 [30]	Turkey	7–18	425	2.4 ± 10.8	− 2.88 ± 8.4	6.0 ± 13.2	3.0 ± 12.0	9.0 ± 13.2	2.4 ± 10.8	4.2 ± 8.4	3.12 ± 0.0
Santoro 2012 [31]	Italy	7–15	291	**4.8 ± 12.0**	x	x	x	x	x	x	x
Hackman and Black 2013 [32]	Scotland	0–21	157	-1.95 ± 14.97	0.00	1.67	5.09	5.06	0.20	4.2	2.00
Zabet 2015 [33]	France	10–18	90	6.44	4.9	-2.2	-2.3	1.2	5.6	15.1	-1.2
Chaumoitre 2017 [34]	France	1–20	1191	*0.72 ± 11.64*	x	x	x	x	x	x	x
Alcina 2018 [35]	Spain	0–18	560	*0.12 (-9.72*,* +11.04)*	x	x	x	x	x	x	x
Alshamran & Offiah 2020 [36]	UK	2–16	186	-0.84 ± 12.6	-1.56 ± 14.16	-5.64 ± 13.56	-11.28 ± 11.88	1.44 ± 13.32	5.88 ± 17.4	-0.6 ± 10.44	x
Current study	UK	10–17	257	0.51 ± 12.43	2.82 ± 11.81	1.33 ± 13.29	-0.29 ± 13.18	-2.71 ± 11.57	4.97 ± 12.54	-0.84 ± 12.64	− 4.19 ± 10.23

* Bold letter means that there are significant differences between mean values of SA and CA (paired t-test analysis). The *italic* letter means that this particular statistical analysis was not reported

Specifically for the female sample, Hackman and Black reported a high and significant correlation between SA and CA (*r* = 0.894; *p* < 0.001), agreeing with our current outcomes (*r* = 0.887; *p* < 0.001). The maximum and minimum difference between SA and CA were similar in the three UK-based studies: the lowest values (delayed SA or underestimation) were − 37 months [32], − 33 months [36] and − 40.17 months (current study) while the highest values (advanced SA or overestimation) were 31 months [32], 36 months [36] and 31.13 months (current study). In this case, Alshamrani and Offiah did not specify if the values were male or female, just global ones. It seems that the present investigation was the one with a maximum underestimation (≈ 3.35 years); whilst Alshamrani and Offiah reported the highest overestimation (≈ 3 years). However, contrasting results were found regarding the MD between SA and CA. Whilst it was negative in both published studies with − 1.95 ± 14.97 months in the Scottish sample [32] and − 0.84 ± 12.6 months in the UK one [36]; it was positive in our current study (0.5 ± 12.43 months).

Outside the UK (Table 5), MD between SA and CA ranged from − 4.68 ± 12.96 months in a German sample [29] to 7.92 ± 13.32 months in a Turkish population [22]. This means that the UK published studies [32, 36] and this current one are within the general range. The UK data also shares a lack of significant differences between the mean SA and mean CA with some studies [23, 30, 33] but not with others [22, 24, 31]. In addition, some literature has reported an overall under-estimation in females when GP is applied [23, 29, 32, 36, 47]. However, other authors also describe the opposite pattern [21, 22, 24, 30, 31, 33–35] which coincides with the present study. Worthy of mention are also the standard deviation values when available in the literature which can range from 10.8 months in one study [30] to 14.97 months in another [32], and this current study is within this interval (12.43 months). General comparison of the global absolute difference between SA and CA was not feasible since only one of the European studies reported it (9.12 months in a French sample [34], a similar value to the 9.96 months shown here).

When data were broken down into age cohorts, some studies showed positive or negative results depending on the age-cohort [30, 33, 36] as in our study; whilst other authors have reported unanimous positive results across all individual age-cohorts [21, 22, 32]. The higher positive discrepancies between SA and CA were reported in 14- [21, 22], 15- [33] and 16-year cohorts in Turkish and French samples, while the 12 year-old cohort [36] reported the higher negative mean value in a UK sample. Over aging of 14 year-olds, as well as 15- and 16 year-olds (Table 5), could be problematic, especially with regard to ages of criminal responsibility in some countries [6, 48].

A combination of different methods should always be used to minimize the margin of error [6, 48, 49]. However, in agreement with AGFAD [50], it is recommended that when reporting the results of age assessments, the following should be stated: the most probable age and the reference standard used, the probability that the estimated chronological age is correct and the most likely minimum and maximum age of the individual undergoing the age assessment.

### Applications

Despite its clinical original goal and the potential skeletal maturation differences between populations, the GP atlas continues to serve law enforcement agencies with age assessment when age is unknown, ambiguous or false [19, 50–54]. Chronological age verification, for example, has been a serious issue in forced marriage in non-legally binding ceremonies, particularly for females [55]. In addition, the threshold of 16-years of age is still important, since it is the age of consent in the UK, meaning “the minimum age of a person with whom another person is legally permitted to engage in sexual activity” [56].

SA estimation has also been applied in sports competitions, whose integrity is based on the validity and accuracy of the ages of participants [11, 57, 58]. For example, skeletal age verification has been performed in FIFA-sponsored international under-17 youth competitions [59]. In addition, several high-profile cases have been reported in age-group sports competitions where birth certificates were allegedly falsified in the Beijing Olympics [60]. However, the existence of population-based differences in maturation rates and the possibility of false positives and negatives make the issue controversial, potentially denying the opportunity to compete for being skeletally mature [10, 11]. Biological maturation and its relationship to chronological age has also implications for exercise programming in youth, conditioning training prescriptions and monitoring exercise adaptations [61].

### Limitations of this study

The timing of skeletal maturational changes, and how they are impacted by socioeconomic factors [32, 41] or population affinity [18, 28], are remarkable for the age estimation process in young individuals [37]. Unfortunately, in the present study, this information was not available. Anthropometric parameters, such as weight and height, were also unknown in the current study, being likely related factors to maturational rates that could affect the estimated SA [62].

Although the lack of repeatability analysis in the present study could seem inappropriate, a study on the University of Copenhagen showed that the direct and naïve use of the GP technique is simple and reproducible, even when applied by non-experienced users [63]. Besides, the MD between the results of two different observers reported to be less than a month and Lin’s concordance correlation coefficient reaching as high as 0.99 [35].

## Conclusions and future investigations

The GP atlas can be applied to the present UK population from the samples analysed here despite the time that has passed since its creation and the secular trends that impact populations. However, biological variation within chronological cohorts must be considered when the atlas is used, specifically in the cohort of those who have reached 14 years of age, according to the MD and MAD between SA and CA which is a matter of concern. Thus, caution should be applied when aging post-pubertal females. Besides, it should be used in combination with other methods as recommended by AGFAD [50] and always considering recently published guidelines for best practices [52]. Relevant medical, family and social history should be also taken into account.

Having said that, critical reviews have advocated that SA estimation cannot be done with sufficient accuracy with the existing methods available [11, 64]. In line with these results, Suri et al. [9] reported that in a normative sample, many 18-year-old girls might not have achieved a GP-based skeletal age of 18 years. In addition, related to the extent ethical concerns about radiation in minors (e.g. plain x-rays of hand-wrist and teeth, clavicle CTs), non-ionising modalities such as MRI and ultrasound have been considered [59, 65–68] and its potential is still being investigated [52, 69–71]. However, further research and validation studies are needed: on the one hand, it is very important to gather more specific information on those factors that can condition an individual’s bone growth and maturation in a given population (e.g. socioeconomic status or medical history), since socioeconomic circumstances can affect nutritional intake, which in turn impacts bone development and ossification rates [72]. On the other hand, further studies on the validity of new methodologies based on the analysis of the ossification of the wrist bones according to the G&P atlas, such as that of Chaumoitre et al. [34], would be of great value. The strengths of this methodology are its very balanced sample across all age groups, which avoids the age mimicry phenomena [73], and the fact that minimum and maximum limits are presented for each age. The latter criteria allows its use in forensic practice, especially in the field of legal age estimation, where the “minimum age principle” [5] is fundamental for a correct assessment of the age of the presumed undocumented minor. In addition, some authors have recently created and applied pioneering tools to generate decision-tree-based methodologies with legal purposes that could improve the legislative classification of challenging individuals [35]. Besides, promising research about how new technologies, such as machine learning and artificial intelligence, could help to enhance age estimation results is being investigated [46, 74, 75].

## Key points

- 1) Age estimation is essential in forensic contexts where data needs to be precise and reliable.
- 2) Overall results show a strong correlation between skeletal and chronological ages in our sample.
- 3) The mean absolute difference (MAD) between skeletal and chronological ages was 9.96 ± 7.44 months.
- 4) The G&P atlas can be accurately applied to the studied UK female sample.
- 5) The G&P methodology must be used along with other age estimation methods.
